# Who Provides? Clinician and Trainee Perspectives on Reproductive Healthcare Access in Vermont

**DOI:** 10.1111/1475-6773.70099

**Published:** 2026-03-19

**Authors:** Varsha Pudi, Kristin Reed, Ian Kent, Claudia Tarrant, Nicholas Khoo, Jonathan Woo, Jeremiah Bates, Oliver Koch, Elizabeth Cote, Charles D. MacLean

**Affiliations:** ^1^ Robert Larner M.D. College of Medicine at the University of Vermont Burlington Vermont USA; ^2^ Vermont Area Health Education Center Burlington Vermont USA; ^3^ Department of Medicine Robert Larner M.D. College of Medicine at the University of Vermont Burlington Vermont USA

**Keywords:** abortion, contraceptives, primary care, reproductive healthcare, women's health, workforce

## Abstract

**Objective:**

To assess Vermont primary care provider (PCP) willingness to expand scope of practice to meet rising reproductive healthcare demands following the 2022 *Dobbs* decision and evaluate the decision's longitudinal implications for workforce development.

**Study Setting and Design:**

We conducted a cross‐sectional, statewide survey of Vermont PCPs and medical trainees in December 2024. Participants were identified via databases maintained by the University of Vermont (UVM) Office of Primary Care and Area Health Education Center Program and the UVM Larner College of Medicine.

**Data Sources and Analytic Sample:**

Data were obtained via survey responses and categorized by professional role, training level, and specialty. Statistical associations were assessed using Chi‐Square tests.

**Principal Findings:**

Over three quarters of Vermont PCPs (77/103, 75%; 95% CI 67%–83%) are willing to expand their scope to ensure safe access to reproductive care. Provision of medication abortion, miscarriage management, and long‐acting reversible contraceptives were identified as areas for scope expansion. Trainees and younger providers were less willing to practice in states with restrictive abortion policies (89/110, 81%; 95% CI 74%–88%) or limited family planning services (90/110, 82%; 95% CI 75%–89%).

**Conclusions:**

Reproductive healthcare policy significantly influences provider career decisions with implications for workforce development in abortion‐restricted states.

## Callout Box

**What is known on this topic?:**

○ The 2022 *Dobbs v. Jackson Women's Health* Supreme Court decision led to increased demand for reproductive healthcare in states where access is legally protected.
○ Some states have enacted legislation to improve access to reproductive healthcare by expanding clinician scope and investing in training programs.
○ Medical trainees have expressed decreased interest in training in states with restrictive reproductive policies, potentially exacerbating provider shortages in underserved regions.

**What this study found?:**

○ Most Vermont primary care providers are willing to expand their scope of practice if necessary to ensure safe access to reproductive healthcare.
○ Younger providers and trainees are less likely to train or practice in states with restrictive reproductive health policies or limited access to family planning services for themselves and their families.
○ Generational disparity exists in terms of clinicians' willingness to expand scope of practice. Educational initiatives that prioritize trainees and younger clinicians may be beneficial in improving reproductive healthcare access.

## 1 Introduction

In June 2022, the *Dobbs v. Jackson Women's Health Organization* Supreme Court decision overturned *Roe v. Wade*, eliminating the federally protected right to abortion care. In the year following *Dobbs*, 783 abortion‐related legal measures were introduced across the United States, underscoring a rapidly evolving legal landscape and the pressing need to understand implications for both patients and clinicians [1].

Ramifications of *Dobbs* include increased interstate travel for abortion, with one in five patients in 2023 traveling out of state, compared to one in ten in 2020 [2]. This has increased the burden in states where abortion remains legal. Planned Parenthood, the largest provider of abortion care nationwide, reported a 12.5% increase in out‐of‐state abortion patients in Vermont, New Hampshire, and Maine, driven by abortion restrictions or longer wait times in home states due to interstate travel surges [3].

Additionally, requests for self‐managed telehealth medication abortion through the nonprofit “Aid Access” increased in all states following *Dobbs* regardless of abortion policy, with the highest increases in states with abortion bans [4]. With increasing demand in states where abortion is legal, it is imperative to assess clinician preparedness and willingness to manage complications of self‐managed medication abortions (SMA) and assume care for out‐of‐state patients. SMA is defined as the termination of a pregnancy using resources outside the formal healthcare system, typically with medications considered to be safe and effective [5]. Clinicians nonetheless play an important role in managing potential SMA‐related complications [6, 7].

*Dobbs* has also raised concerns regarding future workforce development. In abortion‐restricted states post‐*Dobbs*, applicants to OBGYN residency programs declined in two consecutive years—by 10.5% in 2023 [8] and 10.1% in 2024 [9]. Clinicians in abortion‐restricted states face threats of criminal charges, including overwhelming legal fees, loss of licensure, and potential imprisonment [10]. This reality may discourage trainees and clinicians from seeking opportunities in these states, potentially exacerbating existing healthcare disparities.

Given increased demand for reproductive health services, calls to expand PCP reproductive healthcare training have been well‐documented [11]. Historically, PCPs are defined as physicians, Advanced Practice Registered Nurses (APRN), and Physician Assistants in Family Medicine (FM), Primary Care Internal Medicine, and Pediatrics, though some definitions include Women's Health Clinicians because they provide comprehensive care to a significant proportion of women [12, 13]. In this study, we distinguish between PCPs and Women's Health providers.

This study sought to assess Vermont PCPs' preparedness to help meet the growing demand for reproductive healthcare. There is limited research on the role of PCPs in expanding reproductive healthcare access. To address this gap, the goals of this study were three‐fold: (1) assess the current scope of practice of Vermont PCPs regarding the provision of long‐acting reversible contraception (LARC), miscarriage management, and first‐trimester abortion, as well as knowledge of key topics such as abortion complications and clinical course, to identify areas where PCPs are well‐positioned to improve access to comprehensive reproductive healthcare, (2) assess willingness of Vermont PCPs to expand their scope of practice to ensure safe access to medication abortion given increased demand post‐*Dobbs*, and (3) assess how changes in legislation around reproductive healthcare influence workforce development.

## 2 Methods

### 2.1 Study Design

We conducted a statewide electronic survey of Vermont PCPs. A literature review and six key informant interviews with medical students, resident physicians, and clinicians from the University of Vermont (UVM) informed survey development. The interviews assessed clinician scope of reproductive healthcare practice, attitudes toward abortion provision, and policy influence on career decisions. The 34‐question survey was designed to be completed in under 10 minutes, with functionality and timing tested prior to distribution. This study defined fetal viability as greater than or equal to 22 weeks, recognizing this is the lower limit in some guidelines [14]. Survey questions were presented as statements, and responses were measured using 3‐ or 4‐point Likert scales (Figure S1 and Figure S2). The clinician survey was modified for trainees where appropriate.

### 2.2 Participants

An electronic survey was distributed via RedCap to Vermont PCPs. Participants were informed of the investigators and study purpose and were recruited as a convenience sample through the UVM Health Network. The UVM Office of Primary Care and Area Health Education Center Program, which collaborated in this study, maintains a database of Vermont PCPs, updated annually using state licensing data and outreach to practice managers throughout the state. We used a convenience sample of trainees recruited via email lists maintained by the UVM Medical Center and UVM Larner College of Medicine. The trainee survey was distributed to all ACGME‐accredited primary care and OBGYN residency programs in Vermont, and medical students at the state's only medical school. Reminders were sent at Weeks 1 and 2, and at survey closure (Day 18). The study was exempt under Exemption Category 2 45 CFR 46.104(d) (2) by the UVM Institutional Review Board.

### 2.3 Response Rate

Of the 813 invited PCPs, 121 (15%) responded, and of 431 invited trainees, 110 (26%) responded. Nonresponse bias was assessed by comparing aggregate respondent demographics and specialty characteristics with corresponding characteristics from institutional and national data. Individual nonrespondent data were unavailable.

### 2.4 Analytic Strategy

The primary outcome, defined a priori, was the proportion of respondents willing to expand their scope of practice to ensure safe access to reproductive healthcare. Data were analyzed using Stata version 18 [15]. Survey responses were categorized by respondent type (PCPs vs. trainees), and PCPs were further stratified as PCP (non‐Women's Health) versus Women's Health Clinicians, and by generation based on birth year. Categorical variable associations were assessed using Chi‐square tests.

## 3 Results

### 3.1 Participants

The median age of the 121 PCP respondents was 48 years (range 31–79), with 79% women, 17% men, and 4% non‐binary persons. Specialties included 55% FM, 10% IM, 13% Pediatrics, 16% Women's Health, and 7% not listed. Sixty‐eight percent of PCPs were physicians and 32% APRNs. The median trainee age was 26 years (range 23–39), with 62% women, 38% men, and 3% non‐binary persons.

### 3.2 Clinician Survey

#### 3.2.1 Attitudes Toward Abortion Access by Gestational Age

Most respondents supported access to abortion services across gestational ages, with 91% supporting up to 16 weeks and 84% up to age of fetal viability (22 weeks) (Table 1).

**TABLE 1:** Scope of Practice and Attitudes of Vermont PCPs and Women's Health Clinicians^a^.

	Women's health clinicians (*N* = 19)	Other PCPs (*N* = 103)
Support access to abortion services up to 16 weeks' gestation		
Agree or strongly agree	100% (*N* = 19)	89% (*N* = 92)
Neutral	0% (*N* = 0)	4% (*N* = 4)
Disagree or strongly disagree	0% (*N* = 0)	6% (*N* = 6)
Prefer not to answer	0% (*N* = 0)	1% (*N* = 1)
Support access to abortion services up to 22 weeks' gestation		
Agree or strongly agree	89% (*N* = 17)	83% (*N* = 85)
Neutral	5% (*N* = 1)	10% (*N* = 10)
Disagree or strongly disagree	5% (*N* = 1)	8% (*N* = 8)
In my current practice, I provide oral contraceptive management (OCPs)		
Yes	100% (*N* = 19)	91% (*N* = 93)
No	0% (*N* = 0)	10% (*N* = 10)
In my current practice, I provide long‐acting reversible contraception (LARCs)		
Yes	100% (*N* = 19)	52% (*N* = 54)
No	0% (*N* = 0)	48% (*N* = 49)
In my current practice, I counsel newly pregnant individuals about the range of available options		
Yes	100% (*N* = 19)	87% (*N* = 90)
No	0% (*N* = 0)	13% (*N* = 13)
In my current practice, I manage first trimester miscarriages (< 12 weeks)		
Yes	95% (*N* = 18)	25% (*N* = 26)
No	5% (*N* = 1)	75% (*N* = 77)
In my current practice, I provide services for first trimester medical abortion		
Yes	74% (*N* = 14)	13% (*N* = 13)
No	21% (*N* = 4)	86% (*N* = 89)
Prefer not to answer	5% (*N* = 1)	1% (*N* = 1)
I am familiar with the typical clinical course following a medication abortion		
Agree or strongly agree	100% (*N* = 19)	81% (*N* = 83)
Disagree or strongly disagree	0% (*N* = 0)	17% (*N* = 18)
Prefer not to answer	0% (*N* = 0)	2% (*N* = 2)
I am familiar with the common complications following a medication abortion		
Agree or strongly agree	100% (*N* = 19)	79% (*N* = 81)
Disagree or strongly disagree	0% (*N* = 0)	19% (*N* = 20)
Prefer not to answer	0% (*N* = 0)	2% (*N* = 2)
I am interested in learning more about the clinical course following a medication abortion		
Agree or strongly agree	37% (*N* = 7)	84% (*N* = 87)
Disagree or strongly disagree	47% (*N* = 9)	13% (*N* = 13)
Prefer not to answer	16% (*N* = 3)	3% (*N* = 3)
I am willing to expand my scope of practice (within my specialty) if it could help assure safe access to medical abortion		
Agree or strongly agree	84% (*N* = 16)	75% (*N* = 77)
Disagree or strongly disagree	11% (*N* = 2)	19% (*N* = 20)
Prefer not to answer	0% (*N* = 1)	6% (*N* = 6)

^a^ Values are reported as column percentages with raw counts (*N*) in parentheses.

#### 3.2.2 Scope of Reproductive Health Services Provided

All Women's Health Clinicians reported providing oral contraceptive (OCP), LARC, and counseling to newly pregnant individuals about available options (Table 1). Among PCPs, 90% reported offering OCPs, 52% reported offering LARC, and 87% reported counseling newly pregnant patients about available options (Table 1). Ninety‐five percent of Women's Health Clinicians reported providing management for first‐trimester miscarriages (< 12 weeks) and 74% reported providing services for first‐trimester medication abortions. Notably, only 25% of PCPs described managing first‐trimester miscarriages, and 13% reported providing first‐trimester medication abortions.

#### 3.2.3 Familiarity With Medication Abortion and Its Complications

All Women's Health Clinicians and 81% of PCPs reported familiarity with the typical clinical course following medication abortion (Table 1). Generational differences are shown in Table 2. Baby Boomers reported the least familiarity (68%) with the typical course compared with 83%–91% of younger generations (*p* = 0.02, Table 2).

**TABLE 2:** Clinician Attitudes and Willingness to Expand Scope of Practice, by Generation^a^ ^,^ ^f^.

	Baby Boomer (*N* = 25)^b^	Generation X (*N* = 57)^c^	Millennials (*N* = 40)^d^	*P value* ^e^
Familiar with the typical clinical course following a medication abortion (% agree/strongly agree)	68% (*N* = 17)	91% (*N* = 52)	83% (*N* = 33)	0.02
Familiar with the common complications following a medication abortion (% agree/strongly agree)	72% (*N* = 18)	86% (*N* = 49)	83% (*N* = 33)	0.27
Interested in learning more about the clinical course following a medication abortion (% agree/strongly agree)	60% (*N* = 15)	82% (*N* = 47)	80% (*N* = 32)	0.13
Willing to expand my scope of practice if it could help assure safe access to medication abortion (% agree/strongly agree)	52% (*N* = 13)	77% (*N* = 44)	90% (*N* = 36)	0.004

^a^ Includes PCP and Women's Health Clinicians.

^b^ Baby Boomer generation (born 1945–1964).

^c^ Generation X (born 1965–1980).

^d^ Millennials (born 1981–1996).

^e^ *p* values were calculated using Chi‐square tests comparing responses across generations.

^f^ Values are reported as column percentages with raw counts (*N*) in parentheses.

#### 3.2.4 Willingness to Expand Scope of Practice

Among PCPs, 84% reported an interest in learning more about the clinical course following medication abortion (Table 1).

Seventy‐five percent of PCPs and 84% of Women's Health Clinicians reported willingness to expand their scope of practice to ensure safe access to medication abortion (Table 1). Willingness to expand scope varied by generation, with higher willingness among Generation X (1965–1980) (77%) and Millennials (1981–1996) (90%), compared to Baby Boomers (1945–1964) (52%) (*p* = 0.004, Table 2).

#### 3.2.5 Influence of State‐Based Reproductive Policy on Preferred Location of Practice

State‐based reproductive policies significantly influence choice of practice location among younger clinicians (70% of Millennials and 65% of Generation X), compared to 40% of Baby Boomers (*p* < 0.001, Table 3). Younger generations (68% of Generation X and 70% of Millennials) reported that access to comprehensive family planning and reproductive services would influence their preferred practice location, compared to 44% of Baby Boomers (*p* < 0.001, Table 3). Younger generations (79% of Generation X and 88% of Millennials) also reported that they would be less likely to seek future opportunities in states that adopt greater restrictions on reproductive services, compared to 56% of Baby Boomers (*p* < 0.001, Table 3).

**TABLE 3:** State‐based Reproductive Policy Influence on Clinician^a^ and Trainee^b^ Career Decisions^g^.

	Baby Boomer (*N* = 25)^c^	Generation X (*N* = 57)^d^	Millennial (*N* = 40)^e^	Trainee (*N* = 110)^b^	*P value* ^f^
Influence of state‐based reproductive policies on where I prefer to practice					
Large influence	40% (*N* = 10)	65% (*N* = 37)	70% (*N* = 28)	81% (*N* = 89)	
Small influence	12% (*N* = 3)	11% (*N* = 6)	13% (*N* = 5)	15% (*N* = 16)	< 0.001
No influence at all	48% (*N* = 12)	25% (*N* = 14)	18% (*N* = 7)	5% (*N* = 5)	
If a state adopts greater restrictions on reproductive services, my likelihood to seek future opportunities in that state will be					
Less likely					
More likely	56% (*N* = 14)	79% (*N* = 44)	88% (*N* = 35)	91% (*N* = 100)	
No influence	4% (*N* = 1)	4% (*N* = 2)	8% (*N* = 3)	2% (*N* = 2)	< 0.001
Prefer not to answer	40% (*N* = 10)	18% (*N* = 10)	5% (*N* = 2)	7% (*N* = 8)	
Influence of access to the full scope of family planning and reproductive services for me and my family on where I want to practice					
Large influence	44% (*N* = 11)	68% (*N* = 39)	70% (*N* = 28)	82% (*N* = 90)	
Small influence	12% (*N* = 3)	12.% (*N* = 7)	20% (*N* = 8)	12% (*N* = 13)	< 0.001
No influence at all	44% (*N* = 11)	19% (*N* = 11)	10% (*N* = 4)	6% (*N* = 7)	
State‐based legislation around reproductive healthcare influenced where I applied (or will apply) to residency					
Large influence	17% (*N* = 4)	20% (*N* = 9)	44% (*N* = 14)	68% (*N* = 75)	
Small influence	13% (*N* = 3)	13% (*N* = 6)	19% (*N* = 6)	20% (*N* = 22)	< 0.001
No influence at all	70% (*N* = 16)	67% (*N* = 31)	37% (*N* = 12)	12% (*N* = 13)	

^a^ Includes PCP and Women's Health Clinicians.

^b^ Includes medical residents and medical students at the University of Vermont.

^c^ Baby Boomer generation (born 1945–1964).

^d^ Generation X (born 1965–1980).

^e^ Millennials (born 1981–1996).

^f^ *p* values were calculated using Chi‐square tests comparing responses across generations.

^g^ Values are reported as column percentages with raw counts (*N*) in parentheses.

### 3.3 Trainee Survey

#### 3.3.1 Influence of State‐Based Reproductive Policy on Preferred Location of Practice

Among trainees, 68% reported that state‐based reproductive healthcare legislation had strongly influenced the location of their residency applications (Table 3). Eighty‐two percent reported that access to comprehensive family planning and reproductive services for themselves and their families will impact future practice location decisions, and 91% reported being less likely to seek opportunities in states that adopt greater reproductive service restrictions (Table 3).

## 4 Discussion

In this survey, we found that the majority of Vermont primary care providers were willing to expand their scope of practice to ensure safe access to reproductive healthcare, with particularly strong support among younger clinicians. Approximately half of non‐OBGYN PCPs offer LARC, 25% provide miscarriage management, and 13% provide services for medication abortion. With appropriate training and support, PCPs could substantially support reproductive healthcare access by promoting broader provision of these services among providers who do not currently offer them. Finally, our findings suggest that reproductive healthcare legislation affects both trainee and clinician career decisions, with implications for workforce development.

In the post‐*Dobbs* era of increased abortion restrictions, fortifying access to contraception is critical for pregnancy prevention particularly in abortion‐restricted states. Current contraceptive guidelines emphasize the importance of patient‐centered counseling [16]. LARC is among the most effective options, which may be particularly important to some patients, such as those living in rural areas [17]. A study of Title IX‐funded clinics in the Midwest found no difference in the percentage of clinics providing OCPs and injectable contraception between rural and urban areas; however, the percentage of rural clinics offering IUDs, including copper IUDs as emergency contraception, was much lower [18]. Because Vermont is predominantly rural, many patients must travel great distances for specialty services, potentially limiting access to LARC. A survey of PCPs in rural Montana identified lack of training in IUD insertion and removal as the principal barrier to providing LARC [19]. Providing PCP training in LARC provision could potentially increase access for patients living in rural areas.

Several states have launched initiatives to improve reproductive healthcare access by expanding clinician roles and investing in training programs. While some states have historically allowed non‐physician providers to perform abortions, other states have recently revised their laws to expand the abortion provider workforce post‐*Dobbs* [20]. The Pharmacist Abortion Access Project in Washington State is training pharmacists to prescribe abortion medications [20]. Other states have passed legislation to allow more types of clinicians to provide abortions, including physician assistants, nurse practitioners, and nurse midwives, while allocating funding for training initiatives [20]. Our findings suggest that educational initiatives are best targeted toward younger clinicians and trainees. A national survey of FM residency programs conducted 5 months post‐*Dobbs* found that only 19% of responding programs routinely provide medication abortion training and 10% aspiration abortion training, highlighting an area for expansion of abortion provision among PCPs [21]. Our study found that there was substantial interest in learning more about the clinical course following medication abortion among trainees and practicing clinicians.

Delivery of safe and effective reproductive healthcare requires a knowledgeable and prepared workforce. We assessed how changes in state‐based reproductive health policies influence where clinicians and trainees prefer to live, train, and practice. Almost three quarters of clinicians and 91% of trainees indicated that they would be less likely to seek opportunities in states that adopt greater restrictions on reproductive services. Furthermore, other studies found that trainees were more likely to rank residency programs in states without these restrictions, potentially exacerbating existing maternity care deserts [22]. Other factors that may influence willingness to pursue training in abortion‐restricted states include the need to travel out‐of‐state for abortion training and concerns regarding the ability to provide comprehensive reproductive healthcare [22, 23].

Beyond career considerations, trainees reported that having access to the full scope of family planning and reproductive services for themselves and their families significantly influenced their choice of practice location. Some trainees also expressed fears about being forced to carry a pregnancy during training if abortion services were inaccessible [22]. Bernstein and colleagues similarly identified personal access to abortion and family planning services as a key concern for physicians and trainees when considering preferred practice location [24]. Influence of reproductive health policy on older generations was less prominent, likely reflecting clinicians who are at a later stage in their careers and for whom family planning may be less important. Our data indicate that reproductive healthcare policy significantly influences trainee career decisions with implications for workforce development in states with reproductive healthcare restrictions.

Our findings suggest that there are opportunities to expand access to reproductive healthcare through education and training targeted toward the primary care workforce who are interested and willing to expand their scope of practice. Incentives, such as stipends or protected time for continuing education, may encourage PCPs to offer additional services. Local workshops, especially in rural areas, providing training in IUD insertion/removal, alongside didactics on medication abortion and miscarriage management, may improve preparedness to address service gaps and strengthen long‐term workforce development. Beyond Vermont, training content may be tailored to state and local contexts based on existing gaps. By complementing national and state‐level policy interventions with individualized local initiatives, this approach may ameliorate logistical barriers to service provision while supporting sustainable clinician and trainee workforce development. Ensuring reproductive healthcare access in years to come will require coordinated efforts from clinicians, policymakers, and educators to act on recommendations such as expanding scope of practice for PCPs.

## 5 Limitations

This paper has several limitations. First, the response rate was low, but comparable to other response rates of physician surveys. Importantly, there were no statistically significant differences in the sociodemographic characteristics between responders and non‐responders. Second, this study only surveyed providers about their clinical practice patterns; no administrative records or billing data were used to confirm delivery of reproductive healthcare. Third, although the survey reached providers statewide and included trainees at the state's only academic health center, the study's findings may be limited to the unique legal and sociopolitical landscape of Vermont, which was the first state to enact a state constitutional amendment post‐*Dobbs* protecting abortion rights [25].

## 6 Conclusion

Our study suggests that the majority of surveyed Vermont PCPs may be willing to expand their scope of practice to safeguard access to reproductive healthcare. We identified key areas for expansion and further training, such as the provision of medication abortion, miscarriage management, and LARC. Our study identified that trainees and younger clinicians are less likely to seek opportunities in states with restrictive reproductive policies and limited access to comprehensive family planning services, which may exacerbate workforce shortages and reproductive healthcare disparities. Ongoing research will be needed to evaluate the longitudinal impact of *Dobbs* on reproductive healthcare access and workforce development.

## Funding

The authors have nothing to report.

## Conflicts of Interest

The authors declare no conflicts of interest.

## Supporting information


**Figure S1:** Clinician survey.
**Figure S2:** Trainee survey.

## Data Availability

The data that support the findings of this study are available from the corresponding author upon reasonable request.
