# Supplementary material for: Who Provides? Clinician and Trainee Perspectives on Reproductive Healthcare Access in Vermont
Source: Health Serv Res. 2026 Mar 19;61(2):e70099. doi: 10.1111/1475-6773.70099 (PMC13077776; doi:10.1111/1475-6773.70099)
Supplement: Supplementary file 1 — Figure S1: Clinician survey. Figure S2: Trainee survey. [file HESR-61-0-s001.docx]

Supplemental Figure 1. Clinician Survey

*Confidential* *Page* 1 **Reproductive Services and Health Policy**

We are a group of University of Vermont Larner College of Medicine students collaborating with the UVM Office of Primary Care and AHEC Program to better understand the potential impacts of health policy on access to reproductive services.

Reproductive Services is a broad topic and includes access to birth control and family planning, IVF, prenatal testing and care, obstetrical care to support and maintain pregnancy, management of miscarriage, surgical abortion, and medication abortion.

This project examines the influence of reproductive health policy on health care workforce, career decisions, and on the availability of services.

This survey is targeted at trainees (students and residents) and at practicing clinicians in Vermont. Your participation is voluntary, and your responses will be anonymous. You are free to choose "Prefer not to Answer" on any of the questions. We estimate that it will take less than 10 minutes to complete and request that you complete it before November 30th.

We greatly appreciate your time. Thank you for your participation.

UVM LCOM Class of 2027: Jeremiah Bates, Ian Kent, Nicholas Khoo, Oliver Koch, Varsha Pudi, Kristin Reed, Claudia Tarrant, and Jonathan Woo.

With project advisors: Liz Cote, Director, UVM LCOM Office of Primary Care and AHEC Program and Charles MacLean, MD, Professor, Larner College of Medicine

This project has been reviewed and approved by the University of Vermont Institutional Review Board. If you have any questions, please contact Charles MacLean, MD at charles.maclean@uvm.edu

What is your birth year?

__________________________________

What is your gender identity?

Man

Woman

Non-binary

Not listed

Prefer not to answer

(

You may enter "prefer not to answer"

)

__________________________________

What is your professional role?

Medical student

Resident or fellow

Physician

Advanced Practice Registered Nurse or Nurse

Practitioner

Physician Assistant

Not listed

Please specify

__________________________________

What is your specialty?

Family Medicine

Internal Medicine

Pediatrics

Women's Health or OB/GYN

Not listed

In what Vermont county do you practice?

I don't practice in Vermont

Addison

Bennington

Caledonia

Chittenden

Essex

Franklin

Grand Isle

Lamoille

Orange

Orleans

Rutland

Washington

Windham

Windsor

**Specialty choice and LOCATION**

**Decisions about career specialty, the geographic location of a residency program, or about**

**practice location may be influenced by state-level healthcare policy. The following questions**

**are specific to your training, your career choice, and your choices regarding location.**

**Please indicate how much of an influence the following are or were:**

No influence at all

A small influence

A large influence

Prefer not to answer

State-based legislation around

reproductive healthcare

influenced where I applied to

HEALTH PROFESSIONS SCHOOL.

Health policies regarding
reproductive services (at the
federal or state level)
influenced my CHOICE OF SPECIALTY.

State-based legislation around
reproductive healthcare influenced
where I applied to RESIDENCY.
(If applicable)

State-based legislation around
reproductive healthcare influences
where I prefer to PRACTICE.

Access to the full scope of family
planning and reproductive services
FOR ME AND MY FAMILY influences
where I want to practice.

**Please indicate how future state-based reproductive health policies may influence your decisions:**

Less likely to seek MORE likely to seek Changes in Prefer not to answer
opportunities opportunities reproductive health

policy won’t have any

influence on my

decisions

If a state adopts greater
restrictions on reproductive services,
my likelihood to seek future opportunities
in that state will be...

**Personal beliefs, cultural factors, and WORK LOCATION**

**Personal beliefs and cultural factors can influence decisions about where to live, the community of patients to serve, and the health care teams to work with. The following statements focus on preferences for work locations.**

Strongly Disagree Neutral Agree Strongly Prefer not to

Disagree Agree answer

I prefer to work in a SETTING alongside team members who share my views on reproductive services/policies.

I prefer to work in a STATE that

has legislative policies that reflect my

personal beliefs.

I could see myself working as an advocate to influence reproductive policy in a state that has policies or pending legislation that ALIGN with
my beliefs.

I could see myself working as an
advocate to influence reproductive
policy in a state that has policies or
pending legislation that DIFFER from my
beliefs.

**Personal, cultural, or religious beliefs may influence what services healthcare providers are**

**willing to provide (within their typical scope of service).**

Strongly

Disagree

Disagree

Neutral

Agree

Strongly

Agree

Prefer not to

answer

My beliefs limit the reproductive

services I am willing to provide.

If I am unwilling to provide a

reproductive service because of my beliefs, I am willing to refer patients for services.

How important is it for you to
work in a state that has reproductive
 policies that reflect your personal beliefs?

Not at all important

Somewhat important

Very important

**Training and SCOPE OF PRACTICE**

**Primary Care and Women's Health clinicians provide a range of reproductive services. The following questions explore your capacity to provide reproductive services.**

**In my current practice I provide the following reproductive services:**

Yes No Prefer not to answer

Oral contraceptive management

Long-acting reversible contraception (LARC) such as IUD, implantable contraceptives

Counsel a newly pregnant
person about the range of available options

Management of first trimester miscarriage
(< 12 weeks)

First trimester medication abortion

**The 2022 Dobbs v Jackson decision and Primary Care**

**Since the Dobbs v Jackson court decision, access to abortion services has been restricted in many states. Some patients may obtain medications online for medication abortions without clinical supervision. The following questions explore your current knowledge and experience in the area of medication abortion.**

| Strongly disagree | Disagree | Agree | Strongly agree | Prefer not to answer |
| --- | --- | --- | --- | --- |

I am concerned about changes in access to abortion services since the

2022 Dobbs vs Jackson decision.

I am familiar with the TYPICAL
CLINICAL COURSE following a
medication abortion.

I am familiar with the COMMON
COMPLICATIONS following a
medication abortion.

Primary care clinicians (Peds,
FM, IM) should be knowledgeable
about the clinical course following
a medication abortion.

I am interested in learning more
about the clinical course following
a medication abortion.

I am willing to expand my scope
of practice (within my specialty) if
it could help assure safe access
to medication abortion.

**Personal viewpoint**

**Access to reproductive services is a topic that is deeply personal. These questions ask**

**about your personal perspective.**

Strongly

Disagree

Disagree

Neutral

Agree

Strongly

Agree

Prefer not to

answer

I support a patient's access to

reproductive services without

government interference.

Decisions about reproductive
services should be between a patient
and their health care provider.

I support access to abortion
services for victims of sexual abuse,
including rape or incest, without
exceptions.

I support access to abortion
services to protect the life of the pregnant
person, without exceptions.

I support access to abortion
services in cases where the fetus is
not viable, without exceptions.

I support access to abortion
services up to 16 weeks.

I support access to abortion
services up to just below the age of fetal
viability, (approximately 22 weeks).

**We are interested in how familiar you are with some specific legislation.**

Are you familiar with the federal Hyde Amendment?

I have never heard of it

I have heard of it, but would not be able to

explain it

I am familiar with it, and could explain it

Is your work affected by the Hyde Amendment?

Yes

No

I don't know

**Other comments or thoughts**

Do you have any other thoughts or comments that you would like to share?

__________________________________________

Supplemental Figure 2. Trainee Survey

*Confidential* *Page* 1

**Healthcare Workforce Implications of Reproductive Services Health Policies**

We are a group of University of Vermont Larner College of Medicine students collaborating with the UVM Office of Primary Care and AHEC Program to better understand the potential impacts of health policy on access to reproductive services.

Reproductive Services is a broad topic and includes access to birth control and family planning, IVF, prenatal testing and care, obstetrical care to support and maintain pregnancy, management of miscarriage, surgical abortion, and medication abortion.

This project examines the influence of reproductive health policy on health care workforce, career decisions, and on the availability of services.

This survey is targeted at trainees (students and residents) and at practicing clinicians in Vermont. Your participation is voluntary, and your responses will be anonymous. You are free to decline to answer any of the questions. We estimate that it will take less than 5 minutes to complete and request that you complete it before December 20th.

We greatly appreciate your time. Thank you for your participation.

MD Candidates, UVM LCOM Class of 2027: Jeremiah Bates, Ian Kent, Nicholas Khoo, Oliver Koch, Varsha Pudi, Kristin Reed, Claudia Tarrant, and Jonathan Woo

With project advisors: Liz Cote, Director, UVM LCOM Office of Primary Care and AHEC Program and Charles MacLean, MD, Professor, Larner College of Medicine

This project has been reviewed and approved by the University of Vermont Institutional Review Board. If you have any questions, please contact Charles MacLean, MD at charles.maclean@uvm.edu

What is your birth year?

__________________________________

What is your gender identity?

Man

Woman

Non-binary

Not listed

Prefer not to answer

(

You may enter "prefer not to answer"

)

What is your professional role?

Medical student

Resident or fellow

What year are you in medical school?

M1

M2

M3

M4

What residency are you enrolled in (or completed if

Medicine

you are a fellow)?

Surgery (including orthopedics and specialty

surgery)

Pediatrics

Psychiatry

OBGYN

Family Medicine

Neurology

Emergency Medicine

Not Listed

**Decisions about career specialty, the geographic location of a residency program, or about practice location may be influenced by state-level healthcare policy. The following questions are specific to your training, your career choice, and your choices regarding location.**

**Please indicate how much of an influence the following are or were:**

No influence at all A small influence A large influence Prefer not to answer

State-based legislation around
reproductive healthcare influenced
where I applied to MEDICAL SCHOOL

Restrictions on reproductive services are

influencing my CHOICE OF SPECIALTY.

State-based legislation reproductive
healthcare influenced where I applied
(or will apply) to RESIDENCY.

State-based legislation around reproductive
healthcare will influence where I prefer to
PRACTICE.

Access to the full scope of family planning
and reproductive services FOR ME AND
MY FAMILY will influence where I want to
practice.

**Please indicate how future state-based reproductive health policies may influence your decisions:**

Less likely to seek MORE likely to seek Changes in Prefer not to answer
opportunities opportunities reproductive health

policy won’t have any

influence on my

decisions

If a state adopts greater
restrictions on reproductive services,
my likelihood to seek future opportunities
in that state will be...

**Personal beliefs, cultural factors, and WORK LOCATION**

**Personal beliefs and cultural factors can influence decisions about where to live, the community of patients to serve, and the health care teams to work with. The following statements focus on preferences for work locations.**

Strongly Disagree Disagree Neutral Agree Strongly Agree Prefer not to answer

I prefer to work in a SETTING alongside team members who share my views on reproductive services/policies.

I prefer to work in a STATE that
has legislative policies that reflect my
personal beliefs.

I could see myself working as an
advocate to influence reproductive policy in a state
that has policies or pending legislation that ALIGN
with my beliefs.

I could see myself working as an
advocate to influence reproductive policy
in a state that has policies or pending legislation that
DIFFER from my beliefs.

**Personal, cultural, or religious beliefs may influence what services healthcare providers are willing to provide (within their typical scope of service).**

| Strongly disagree | Disagree | Agree | Strongly agree | Prefer not to answer |
| --- | --- | --- | --- | --- |

My beliefs limit the reproductive
services I am willing to provide.

If I am unwilling to provide a
reproductive service because of
my personal beliefs, I am willing
to refer patients for services.

How important is it for you to
work in a state that has reproductive
 policies that reflect your personal beliefs?

Not at all important

Somewhat important

Very important

**Training and SCOPE OF PRACTICE**

**Primary Care and Women's Health clinicians provide a range of reproductive services. The following questions explore your capacity to provide reproductive services.**

**At this point in my training, I feel prepared to provide the following reproductive services:**

Yes No Prefer not to answer

Oral contraceptive management

Long-acting reversible contraception (LARC)
such as IUD, implantable contraceptives

Counsel a newly pregnant person
about the range of available options

**Since the Dobbs v Jackson court decision, access to abortion services has been restricted in**

**many states.**

Strongly

disagree

Disagree

Neutral

Agree

Strongly

agree

Prefer not to

answer

I am concerned about changes

in access to abortion services

since the 2022 Dobbs vs Jackson

decision.

**Personal viewpoint**

**Access to reproductive services is a topic that is deeply personal. These questions ask about**

**your personal perspective.**

Strongly

disagree

Disagree

Neutral

Agree

Strongly

agree

Prefer not to

answer

I support a patient's access to

reproductive services without

government interference.

Decisions about reproductive
services should be between a
patient and their health care provider.

I support access to abortion
services for victims of sexual abuse,
including rape or incest,
without exceptions.

I support access to abortion
services to protect the life of the
pregnant person, without
exceptions.

I support access to abortion
services in cases where the fetus
 is not viable, without exceptions.

I support access to abortion services up to 16 weeks.

I support access to abortion services up to just below the age of fetal viability,
(approximately 22 weeks).

Are you familiar with the Hyde
Amendment regarding federal
funding for reproductive services?

Never heard of it

Have heard of it, but cannot

explain it

Heard of it and can explain it

**Other comments or thoughts**

Please let us know if you have any other comments or perspectives here:

__________________________________________
